# Enzyme replacement therapy and/or hematopoietic stem cell transplantation at diagnosis in patients with mucopolysaccharidosis type I: results of a European consensus procedure

**DOI:** 10.1186/1750-1172-6-55

**Published:** 2011-08-10

**Authors:** Minke H de Ru, Jaap J Boelens, Anibh M Das, Simon A Jones, Johanna H van der Lee, Nizar Mahlaoui, Eugen Mengel, Martin Offringa, Anne O'Meara, Rossella Parini, Attilio Rovelli, Karl-Walter Sykora, Vassili Valayannopoulos, Ashok Vellodi, Robert F Wynn, Frits A Wijburg

**Affiliations:** 1 Department of Pediatrics and Amsterdam Lysosome Center 'Sphinx', Academic Medical Center, University of Amsterdam, Amsterdam, The Netherlands; 2 Department of Pediatrics, Pediatric Blood and Marrow Transplantation Program, University Medical Center Utrecht, Utrecht, The Netherlands; 3 Department of Pediatrics, Clinic for Kidney-, Liver- and Metabolic diseases, Hannover Medical School, Hannover, Germany; 4 Genetic Medicine, Manchester Academic Health Science Centre, University of Manchester, Central Manchester University Hospitals NHS Foundation Trust, St Mary's Hospital, Manchester, UK; 5 Department of Pediatric Clinical Epidemiology, Academic Medical Center, University of Amsterdam, Amsterdam, The Netherlands; 6 Pediatric Immunology-Hematology Unit, Necker-Enfants Malades Hospital, Assistance Publique-Hôpitaux de Paris (AP-HP), Paris, France; 7 Villa Metabolica, Children's Hospital, Gutenberg-University, Mainz, Germany; 8 Department of Haematology/Oncology, Our Lady's Children's Hospital, Dublin, Ireland; 9 Rare Metabolic Diseases Unit, Department of Pediatrics, San Gerardo Hospital, Monza, Italy; 10 Bone Marrow Transplant Unit, Department of Pediatrics, San Gerardo Hospital, Monza, Italy; 11 Department of Pediatric Hematology/Oncology, Hannover Medical School, Hannover, Germany; 12 Reference Center for Inherited Metabolic Disorders, Necker-Enfants Malades Hospital, Paris, France; 13 Metabolic Unit, Great Ormond Street Hospital for Children NHS Trust, London, UK; 14 Department of Blood and Marrow Transplant, Royal Manchester Children's Hospital, Manchester, UK

## Abstract

**Background:**

Mucopolysaccharidosis type I (MPS I) is a lysosomal storage disorder that results in the accumulation of glycosaminoglycans causing progressive multi-organ dysfunction. Its clinical spectrum is very broad and varies from the severe Hurler phenotype (MPS I-H) which is characterized by early and progressive central nervous system (CNS) involvement to the attenuated Scheie phenotype (MPS I-S) with no CNS involvement. Indication, optimal timing, safety and efficacy of the two available treatment options for MPS I, enzyme replacement therapy (ERT) and hematopoietic stem cell transplantation (HSCT), are subject to continuing debate. A European consensus procedure was organized to reach consensus about the use of these two treatment strategies.

**Methods:**

A panel of specialists, including 8 specialists for metabolic disorders and 7 bone marrow transplant physicians, all with acknowledged expertise in MPS I, participated in a modified Delphi process to develop consensus-based statements on MPS I treatment. Fifteen MPS I case histories were used to initiate the discussion and to anchor decisions around either treatment mode. Before and at the meeting all experts gave their opinion on the cases (YES/NO transplantation) and reasons for their decisions were collected. A set of draft statements on MPS I treatment options composed by a planning committee were discussed and revised during the meeting until full consensus.

**Results:**

Full consensus was reached on several important issues, including the following: 1) The preferred treatment for patients with MPS I-H diagnosed before age 2.5 yrs is HSCT; 2) In individual patients with an intermediate phenotype HSCT may be considered if there is a suitable donor. However, there are no data on efficacy of HSCT in patients with this phenotype; 3) All MPS I patients including those who have not been transplanted or whose graft has failed may benefit significantly from ERT; 4) ERT should be started at diagnosis and may be of value in patients awaiting HSCT.

**Conclusions:**

This multidisciplinary consensus procedure yielded consensus on the main issues related to therapeutic choices and research for MPS I. This is an important step towards an international, collaborative approach, the only way to obtain useful evidence in rare diseases.

## Background

Mucopolysaccharidosis type I (MPS I) is a lysosomal storage disorder caused by a deficiency of the enzyme α-L-iduronidase (IDUA) [1] and has an estimated prevalence of 0.69 to 3.8 per 100,000 live births [2,3]. Inheritance is autosomal recessive and more than 100 different mutations in the *IDUA *gene have been described [4]. Reduced or absent IDUA activity results in accumulation of the glycosaminoglycans (GAGs) heparan and dermatan sulfate throughout the body which leads to widespread cellular, tissue, and organ dysfunction, and thus progressive disease.

Historically, MPS I is delineated into three disease phenotypes based on clinical presentation: MPS I-H (Hurler syndrome, severe phenotype), MPS I-H/S (Hurler-Scheie syndrome, intermediate phenotype) and MPS I-S (Scheie syndrome, attenuated phenotype). Patients with MPS I-H have an early-onset, rapidly progressive disease with CNS involvement, which, if left untreated, results in early death, usually within the first two decades of life. Patients with MPS I-S have a much slower disease progression without clinical involvement of the CNS and with a near normal life expectancy. Other common MPS I features include coarse facial features, hepatosplenomegaly, cardiac disease, joint stiffness, skeletal deformities and corneal clouding, all with highly variable severity [1,5].

Currently, the phenotype is recognized as a continuous spectrum, ranging from severe, with progressive involvement of the central nervous system resulting in cognitive decline, to attenuated, without clinical involvement of the central nervous system. Delineation of the different MPS I phenotypes can be difficult and is based on the age of presentation, rate of progression and the genotype of the patient [6].

Intravenous enzyme replacement therapy (ERT) with laronidase (recombinant IDUA, Aldurazyme^®^) has proven to be safe and effective in MPS I patients across a wide range of ages and disease severity, significantly ameliorating somatic disease [7-12]. Laronidase is unable to cross the blood brain barrier in significant quantity, at least in the recommended dose (100 IU/kg/week) [13], and will therefore not prevent cognitive decline in patients with MPS I-H. A recent report showed that early and higher-dose intravenous ERT can reduce lysosomal storage of GAGs in the brain in dogs with MPS I [14]. However, this has not been studied in humans.

Hematopoietic stem cell transplantation (HSCT) is the treatment of choice in the more severely affected patients with CNS involvement. It has been shown to preserve intellectual development when performed early in the course of disease [15,16]. Currently, HSCT is considered to be indicated for MPS I-H patients under the age of 2 years and with no or only moderate cognitive impairment (Developmental Quotient (DQ) > 70) [6]. HSCT carries considerable risks for procedure-related morbidity and mortality. However, in recent years, transplant related mortality has declined and the rate of engraftment has improved, resulting in survival rates with donor cell engraftment of over 90% [17]. In addition, laronidase is increasingly used as an adjuvant treatment before HSCT to improve the pre-transplant clinical condition [6,17].

With increasing insight in the efficacy and limitations of both disease-modifying treatments, it is important to update guidelines for treatment. Preferably, these guidelines are based on high-level empirical evidence. However, due to the extreme rarity of the disease, until now only one randomized controlled trial has been performed, which was on the efficacy of laronidase [8]. Efficacy of HSCT in improving clinical outcome has only been assessed by retrospective analyses of cohorts, and no trials have been done comparing both treatment modalities.

In order to update management guidelines, combining all available published evidence with current expert knowledge, a European consensus procedure was organized with the participation of a group of pediatricians experienced in treatment of patients with MPS I, and comprising both metabolic specialists and bone marrow transplant specialists. The goal was to obtain consensus on crucial issues related to the two different therapeutic modalities in MPS I in order to provide clinical guidelines.

## Methods

A modified Delphi technique was used. This methodology, developed by the Rand Corporation/University of California, Los Angeles (UCLA) [18], is based on the original Delphi process [19], which has been widely used to achieve consensus on a specific issue, and is increasingly used for the development of clinical guidelines when there is insufficient evidence [20,21].

To initiate the process, a literature review was performed by two members of a planning committee (MHdR, FAW), to gather the best available published evidence on ERT and HSCT in MPS I. An electronic search was conducted using Pubmed and EMBASE to identify all relevant articles. Criteria used as key words were: mucopolysaccharidosis I, MPS I, Aldurazyme, Laronidase, alpha-L-iduronidase, enzyme replacement therapy, bone marrow transplantation and hematopoietic stem cell transplantation.

Secondly, a European expert panel was composed, consisting of 8 pediatricians for metabolic disorders and 7 bone marrow transplant physicians, all with acknowledged expertise in the field of MPS I treatment and research. In addition, 15 MPS I case histories were collected by two members of the planning committee (MHdR, FAW), based on existing MPS I patients. The case histories contained the patients' age at diagnosis, signs and symptoms related to MPS I at the time of diagnosis, the results of relevant diagnostic tests and, if available, a picture of the patient at the time of diagnosis. Genotypes, although they can be used to predict disease severity in some patients, were not included in the case histories, and treatment decisions were therefore based on clinical signs and symptoms. This was done as the planning committee felt that genotype-phenotype correlations are still limited, and as there is already full agreement on the predictive value of a few (nonsense) mutations. The case histories were chosen to represent the full range of the MPS I phenotypic spectrum. Age at diagnosis ranged from 5 months to 9 years.

The 15 MPS I case histories were sent to each of the panel members ('written' round 1), who were asked to give their opinion on the choice of treatment (deciding 'YES' or 'NO' in favor of HSCT) for each patient based on their clinical judgment, and to explain their decision by reporting the most important reasons on which their decision was based as well as the perceived issues complicating the decision in that particular case.

Draft statements on MPS I treatment options, based on the literature review and personal experience, were composed by members of the planning committee (FAW, JJB, SAJ, RFW) (Table 1).

**Table 1:** Draft statements composed by the planning committee

Statement	
**1**.	All patients should be genotyped at diagnosis as this may help in (future) decision-making on therapeutic strategies.

**2**.	Patients homozygous or compound heterozygous for mutations clearly associated with MPS I-H (e.g. W402X, Q70X) should be referred for HSCT.

**3**.	Patients diagnosed before the age of 2.5 years based on clinical signs and symptoms compatible with MPS I-H (i.e. early kyphosis and/or characteristic facial features and/or CNS-involvement) should be referred for HSCT.

**4**.	HSCT is more successful if performed early and should probably be done after the age of 3 months, as soon as a suitable donor is available.

**5**.	All patients with MPS I should be tested by an experienced child (neuro-)psychologist for developmental quotient at diagnosis. Tests should be adapted for physical limitations (e.g. auditory or visual handicaps). If there is significant developmental delay (DQ < 70) before transplant, the outcome of HSCT on final IQ is likely to be limited. Not performing HSCT in these patients should be considered as an option.

**6**.	There is yet no evidence that HSCT is the optimal treatment for patients with MPS I-H/S and MPS I-S (patients diagnosed on the basis of first significant clinical signs and symptoms > 2.5 years and a genotype not indicating MPS I-H). A randomized controlled trial should elucidate if HSCT is the optimal strategy for these patients.

**7**.	All patients that are not transplanted may benefit significantly from ERT.

**8**.	As the efficacy of ERT improves if initiated at an early age, ERT should be started at diagnosis.

**9**.	There is no evidence that a dose other than the recommended dose (100 IU/kg weekly) of alpha-L-iduronidase is superior. A randomized controlled trial will be the best strategy to elucidate the optimal dose.

**10**.	Patients who will be/are referred for HSCT may benefit from ERT before HSCT as this can improve the clinical condition of the patient.

**11**.	There is no evidence that ERT before HSCT interferes with engraftment.

**12**.	There is no evidence that ERT after a successful HSCT will improve clinical outcome.

These are NOT the final statements (see results).

Subsequently, the panel members met during a one-day face-to-face meeting in Amsterdam, the Netherlands, chaired by an experienced moderator (MO), not involved in MPS I treatment. At the start of the meeting, the same 15 MPS I case histories were presented to the panel members again, yet in a randomly different order, to determine intra-observer reproducibility ('written' round 2). This time no reasons for, or issues complicating decisions were collected. Intra-observer reproducibility was quantified by Cohen's kappa [22]. This is a measure of agreement which corrects for agreement by chance. Kappa values may vary from 0 (complete disagreement) to 1 (complete agreement). The interpretation of kappa is arbitrary. Generally, a kappa value of 0.61-0.80 is considered to denote "good agreement" [23]. Wilcoxon's signed rank test was used to compare the proportions of pediatricians for metabolic disorders and bone marrow transplant physicians in favor of HSCT per round.

The results of round 1, including the reported issues used for, or complicating, decision making, were used as a starting point for subsequent discussion. The most appropriate treatment modality for each patient was discussed in detail with a particular focus on areas of controversies. Decisions made in the first round were compared with the decisions made in the second round. The aim of this discussion was to gather all relevant issues related to the decision making processes on treatment decisions (i.e. installing ERT and/or HSCT) in MPS I. Finally, the draft consensus statements were discussed and revised until full consensus was reached, and a treatment algorithm for MPS I patients was composed.

## Results

**Figure 1:**
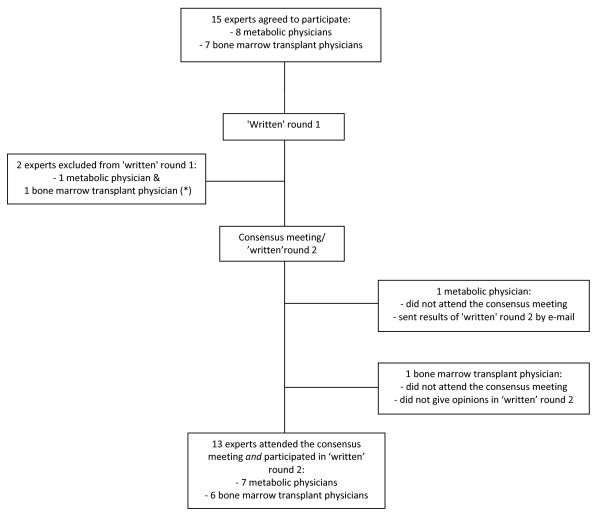
**Flow chart of the consensus process**. (*)Two experts were excluded from 'written' round 1, because the opinions on their choice of treatment in round 1 were not obtained individually.

All 15 invited experts participated in 'written' round 1 (8 metabolic physicians and 7 bone marrow transplant physicians), and 14 participated in 'written' round 2 (8 metabolic physicians and 6 bone marrow transplant physicians). Since it was found out later that 2 experts had both replied to 'written' round 1 after ample discussion, their responses were excluded from the results. Two of the experts (1 metabolic physician and 1 bone marrow transplant physician) could not attend the face-to-face meeting, but one of them responded by e-mail to 'written' round 2. 13 experts attended the face-to-face meeting (7 metabolic physicians and 6 bone marrow transplant physicians), and responses to 'written' round 2 were obtained from 14 experts. A flow chart of the process is shown in Figure 1.

The number of experts in favor of HSCT varied between the 15 case histories from 1 to 13 out of 13 in round 1 and 1 to 14 out of 14 in round 2. The median [range] number of experts in favor of HSCT for all cases was 7 [1-13] and 4 [1-14] in rounds 1 and 2, respectively (Table 2). The most frequently reported issues considered 'important' or 'complicating' for deciding on HSCT are ranked in Tables 3 and 4.

**Table 2:** Expert opinions given in rounds 1 and 2 (per patient) divided by physician specialty

	Round 1					Round 2			
**Patient**	**'YES' for HSCT**		**'NO' for HSCT**		**Missing**	**'YES' for HSCT**		**'NO' for HSCT**	
	
	**MP**	**TP**	**MP**	**TP**		**MP**	**TP**	**MP**	**TP**

**1**.	0	2	7	4		0	2	8	4

**2**.	3	4	4	2		1	3	7	3

**3**.	7	6	0	0		8	6	0	0

**4**.	3	4	4	2		4	3	4	3

**5**.	6	6	1	0		8	6	0	0

**6**.	6	5	1	1		8	6	0	0

**7**.	2	5	5	1		1	2	7	4

**8**.	2	5	5	1		3	4	5	2

**9**.	0	1	7	5		0	1	8	5

**10**.	0	1	7	4	1	0	1	8	5

**11**.	0	1	7	5		0	1	8	5

**12**.	5	4	2	2		6	4	2	2

**13**.	1	4	6	2		1	2	7	4

**14**.	1	3	6	3		0	4	8	2

**15**.	6	5	1	1		7	5	1	1

**Median**	2	4	5	2		1	3	7	3

MP = Metabolic Physician

TP = Transplant Physician.

**Table 3:** Top 5 most frequently reported important criteria to choose 'YES' or 'NO' for HSCT

**Important for 'YES' for HSCT**

1. Developmental delay^1^

2. Young age

3. Severe phenotype

4. Kyphosis

5. Likely to benefit

**Important for 'NO' for HSCT**

1. Milder phenotype

2. Older age

3. Normal development

4. Developmental delay^1^

5. Mainly joint stiffness/late diagnosis

^1 ^Also stated as 'low IQ' or 'CNS-involvement'.

**Table 4:** Top 5 most frequently reported complicating issues to choose 'YES' or 'NO' for HSCT

**Complicating for 'YES' for HSCT**

1. Developmental delay^1^

2. Older age

3. Cardiomyopathy

4. ERT prior to HSCT desirable

5. Uncertain (CNS) prognosis

**Complicating for 'NO' for HSCT**

1. ERT prior to HSCT might improve symptoms

2. Developmental delay^1^

3. Long-term course unknown

4. Unknown role for HSCT in milder phenotype

5. Early diagnosis

^1 ^Also stated as 'low IQ' or 'CNS-involvement'.

The intra-observer median [range] kappa was 0.73 [0.09 - 1]; 10 physicians had a kappa > 0.70. The proportion of transplant specialists in favor of HSCT (median 0.7 and 0.5 in rounds 1 and 2, respectively) was significantly higher than the proportion of metabolic pediatricians in favor of HSCT (median 0.3 and 0.1 in rounds 1 and 2, respectively) for all 15 MPS I case histories (p = 0.003 and p = 0.007, respectively).

**Figure 2:**
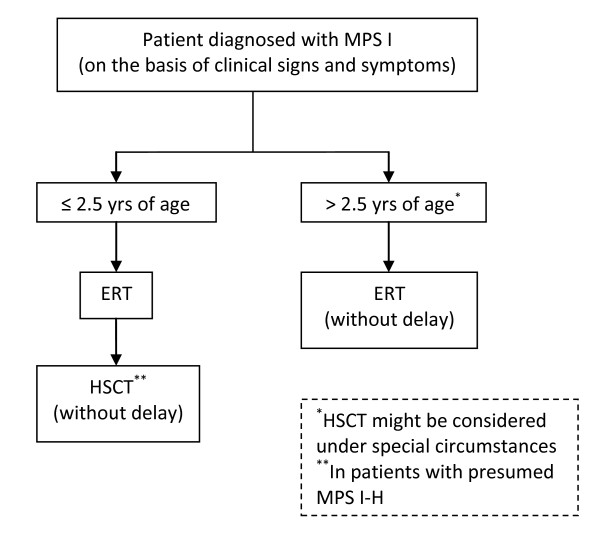
**Treatment algorithm for MPS I patients**.

The discussion and revision of the draft statements, composed before the meeting by the planning committee (Table 1) led to full consensus, indicated by expression of agreement by all participants, on the 9 statements described below. In addition, a treatment algorithm for patients with a diagnosis of MPS I, modified from the algorithm presented by Muenzer et al. [6], was composed on the basis of the final statements (Figure 2) and first steps towards a collaborative research agenda were made.

### 1. Decisions on selection of disease modifying treatment for a patient with MPS I should be made within a team. This team should include at least a metabolic physician, a bone marrow transplant physician and a neuropsychologist, all with expertise in MPS I

#### Rationale

As knowledge on indication, optimal timing and efficacy of the two disease modifying treatment strategies in MPS I (ERT and HSCT) is subject to continuous change, and as management decisions involve judgment calls and preferences, the choice of therapy for any given patient with MPS I should be discussed by a team of experts. Only by this dynamic process patients can be offered an optimal therapeutic strategy (*expert consensus*).

### 2. The genotype should be established at diagnosis in all patients with MPS I as this may help in decision making on therapeutic strategies

#### Rationale

Genotype-phenotype correlations are still limited in MPS I, except for a small number of mutations which invariably result in MPS I-H if present in homozygous or compound heterozygous combination [4], and few mutations which reliably predict MPS I-S [24]. However, new reports [25] and accumulating (unpublished) data from the MPS I Registry, an observational program designed to collect data of MPS I patients with the primary purpose to study the clinical onset, symptoms, and outcomes of these patients [5], show that the predictive value of the genotype in MPS I may increase substantially in the (near) future (*expert consensus*). This will help in forming the decision on the optimal strategy in individual patients, and may eventually become indispensable for future introduction of newborn screening for MPS I (*expert consensus*). As polymorphic variations within the *IDUA *gene may modify the expression of mutations [26], new studies are needed to determine the role of these polymorphic variations (*expert consensus*).

### 3. The preferred treatment strategy for patients diagnosed before the age of 2.5 years and with presumed MPS I-H (presence of clinical signs and symptoms compatible with MPS I-H, i.e. phenotypic diagnosis based on clinical expertise), and/or homozygosity or compound heterozygosity for mutations exclusively associated with the severe phenotype is HSCT

#### Rationale

ERT is not effective in preventing neurocognitive decline in patients with MPS I-H, as the recombinant enzyme will not cross the blood-brain barrier in sufficient quantity [7-11]. Early treatment by HSCT may preserve cognition and may ameliorate or prevent several of the somatic manifestations of the disease [15,16,27-30]. In addition, recent advances in chemotherapeutic conditioning and donor selection have significantly improved the outcome of this procedure [29,30]. Therefore, HSCT is the preferred treatment for patients with MPS I-H. Since phenotypic variability exists even within the severe phenotype, the previously set upper age limit of 2 years for HSCT cannot be used as a strict criterion, and a limit of '2.5 years' is more justified *(expert consensus)*.

### 4. HSCT in patients with MPS I-H is more successful if performed early, and should be performed as soon as the somatic condition allows for the procedure

#### Rationale

There appears to be a time lag between full hematopoietic engraftment and the start of stabilization of cognitive decline. Early transplantation, before overt developmental deterioration has become clear, probably allows the highest chances of better outcome in intellectual functions (*expert consensus*). A younger age at transplantation is an individual predictor for higher neutrophil recovery after HSCT. Also, younger children tend to receive favorable higher cell doses from the donor graft, since they are smaller. A shorter interval between diagnosis and transplantation positively influences the outcome [29,30].

### 5. Children with MPS I and advanced CNS-disease (i.e. DQ < 70) are less likely to benefit from transplant. There should however not be an undue reliance on developmental assessment in young children with MPS I

#### Rationale

Poor neurological outcome after HSCT is related to lower IQ/DQ before transplantation [16]. Developmental delay in an individual child might be related to severe somatic disease such as joint stiffness, hearing deficits and visual handicaps. A well-trained neuropsychologist with experience in testing children with MPS I-H should take these factors into account when assessing the developmental level in a patient (*expert consensus*). The decision whether to transplant should subsequently be made by the team (statement 1).

### 6. In any individual patient with MPS I-H/S and CNS-involvement, HSCT may be considered if there is a suitable donor. However, at present there are no data on the efficacy of HSCT in patients with this phenotype

#### Rationale

Patients with MPS I-H/S may display symptoms of CNS involvement of variable severity [5,31]. However, the long-term risk for progressive CNS-disease in these attenuated patients is not known (*expert consensus*). Since transplant related mortality has declined significantly and as HSCT has been demonstrated to be able to halt cognitive decline in patients with MPS I-H, HSCT might also be considered as therapeutic strategy if patients with MPS I-H/S display progressive neurocognitive involvement, but only if a suitable donor is available (*expert consensus*). In order to study potential benefits of HSCT in patients with MPS I-H/S, data on the outcome should be collected in a coordinated multi-centered fashion (*expert consensus*).

### 7. All MPS I patients (including those who have not been transplanted or whose graft has failed) may benefit significantly from ERT as this will ameliorate several somatic symptoms of the disease

#### Rationale

Clinical trials have demonstrated that ERT is safe and effective in patients with MPS I across a wide range of ages (young children to adults) and phenotypes (severe and attenuated) [7-12]. Long-term ERT effectively treats several somatic signs and symptoms of MPS I, resulting in improved health-related quality of life (HRQOL) [9,11].

### 8. As the efficacy of ERT improves when initiated at an early age, ERT should be started at diagnosis

#### Rationale

End organ damage, associated with progressive GAG accumulation, may worsen and become irreversible in untreated MPS I patients (*expert consensus*). Therefore, initiation of treatment early in the disease course is likely required to prevent and/or minimize irreversible damage at least in symptomatic patients (*expert consensus*). A case history on siblings started on ERT at different ages supports this statement [32].

### 9. Patients who are referred for HSCT may benefit from ERT before HSCT as this can improve the clinical condition of the patient

#### Rationale

The clinical condition of a patient at the time of transplantation will influence the morbidity and mortality of the HSCT (*expert consensus*). Since ERT has shown to result in a significant and relatively rapid improvement of respiratory and cardiovascular function [7-11], ERT may ameliorate the pre-transplant condition of a patient. It was shown that ERT prior to HSCT is well tolerated and may significantly improve the pre-transplant condition in selected patients [33-36]. In addition, there is evidence that ERT does not negatively influence engraftment [33-36]. However, initiating ERT should not delay the transplant (*expert consensus*).

## Discussion

This European consensus project was organized to develop recommendations for decision making on the two currently available disease-modifying treatment options for MPS I patients. This is important, because it concerns a debilitating condition, involves treatment modalities that are associated with health risks and considerable financial costs, and there is uncertainty among practitioners on the optimal management strategies in the various different phenotypic subtypes. We chose a modified Delphi process to provide a means to combine the very limited scientific evidence available on this subject with expert opinion, and reached consensus on 9 key areas for decisions and the underlying rationale for the guidance statements. To our knowledge, this is the first consensus project including both pediatricians for metabolic disorders and bone marrow transplant physicians, all experienced in the treatment of MPS I patients.

Over the last three decades, more than 500 HSCTs for children with MPS I-H have been performed worldwide [17]. In recent years, transplant related mortality has declined considerably with survival rates exceeding 90%. Engraftment has improved equally. International guidelines, which include the use of unrelated cord blood as cell source, therapeutic drug monitoring of Busulfan and short interval between diagnosis and transplantation are the most important causes for these improvements [17,29,30]. As a result, HSCT might be considered as a therapeutic strategy for patients with MPS I-H/S and progressive neurocognitive involvement (i.e. the more severe MPS I-H/S phenotype). However, convincing evidence is needed to support the use of HSCT in this subgroup of MPS I patients. Unfortunately, there is hardly any knowledge on the natural course of the disease in patients with the intermediate phenotype. Until such information becomes available, HSCT in these patients should only be performed if the risk-benefit ratio is considered to be favorable and an optimal donor is available.

Laronidase received marketing approval in the USA and Europe in 2003. Several clinical trials, including one randomized controlled trial, have demonstrated its safety and efficacy for the treatment of non-neurological symptoms of the disease [7-11], and currently over 600 patients with MPS I receive or have received laronidase (data presented at the MPS I Registry International Board of Advisors meeting, Istanbul, Turkey, August, 2010, from the MPS I Registry, Genzyme Corp., USA). Treatment with laronidase should be started at diagnosis in all symptomatic MPS I patients and laronidase may also improve the pre-transplant condition in patients who are awaiting HSCT.

There was complete disagreement (7 in favor of HSCT vs. 6 not in favor of HSCT) on the preferred treatment strategy in round 1, being HSCT or no HSCT, in 4 out of the 15 MPS I case histories (Table 2), which underscores the difficulties in choosing the most appropriate course of action, even among experts. All these cases were classified as intermediate, MPS I-H/S, phenotype during the discussion. Despite the initial differences of opinion between metabolic physicians and bone marrow transplant physicians on preferred treatment, with transplant physicians more often favoring HSCT, elicitation and discussion of the underlying motives and concerns led to full consensus regarding the final statements and a joint formulation of the rationale for these statements.

Based on expert experience, the panel reached consensus that the age limit for HSCT should not be set too low, as considerable preservation of CNS functions may still be achieved in patients with MPS I-H if a successful HSCT is done even after 2 years of age. In addition, the panel agreed that reliable testing of cognitive development (DQ/IQ) in young patients with MPS I can be very difficult due to symptoms of the disease, including restricted motor performance. The consensus statement on this issue (statement 5) was therefore formulated with caution, and DQ/IQ is not included as a criterion in the algorithm. The algorithm cannot replace clinician judgment, and team decisions on treatment should always be considered on a patient by patient basis. Yet, the issues reported in Tables 3 and 4 will play an important role in this process.

All participants agreed that further studies are needed to create evidence based protocols on the treatment of MPS I patients in the future. Due to the extreme rarity of the disease, it is essential to try to gather further evidence in an international, interdisciplinary, collaborative approach. As formulated by the panel, future research should have a particular focus on the long-term neurocognitive outcome of patients with MPS I-H/S, and on the potential role of HSCT in the treatment of this phenotypic group. With the prospects of newborn screening for MPS I [37], reliable tools to predict the clinical phenotype in presymptomatic children are urgently needed. In this respect, a better understanding of genotype-phenotype correlations in MPS I, including the potential role of modifying polymorphisms [26] and the possible role of biomarkers in predicting disease severity are essential. The consensus procedure did not include discussion on possible indications for laronidase in MPS I patients after HSCT. Experience with ERT in these patients is still very limited [38], and further studies are needed to elucidate the role of laronidase in this group.

## Conclusions

We conclude that the modified Delphi consensus procedure used to obtain statements on crucial issues related to decisions on treatment options in newly diagnosed MPS I patients was instrumental in obtaining 9 important consensus statements. Further collaborative studies will help to optimize the clinical decision making process, which will lead to improvements in life expectancy and quality of life in MPS I patients.

## Competing interests

AMD, EM, AV, and FAW received research and travelling grants and reimbursement of expenses and honoraria for lectures on lysosomal storage diseases from Genzyme Corporation. SAJ, NM, RP, and VV received travelling grants and honoraria for lectures on lysosomal storage diseases from Genzyme Corporation. MHdR, JJB, JHL, MO, AOM, AR, KWS, and RFW declare no competing interests.

## Authors' contributions

JJB, SAJ, RFW, and FAW were part of the planning committee, composed the draft statements, and assisted in writing the first draft of the manuscript. MHdR and FAW organized the consensus meeting, and were involved in the methodological process. MHdR assisted in composing the case histories, wrote down the first draft of the manuscript, and assisted in the statistical analysis. MO was the moderator, assisted in writing the first draft of the manuscript, assisted in the statistical analysis, and was involved in the methodological process. JHL assisted in organizing the consensus meeting, was involved in the methodological process, carried out the statistical analysis, and assisted in writing the first draft of the manuscript. JJB, AMD, SAJ, NM, EM, AOM, RP, AR, KWS, VV, AV, RFW, and FAW participated in 'written' rounds 1 and 2, and the face-to-face consensus meeting. All authors read and approved the final manuscript.
